# Evolution of Gold and Iron Oxide Nanoparticles in Conjugates with Methotrexate: Synthesis and Anticancer Effects

**DOI:** 10.3390/ma16083238

**Published:** 2023-04-19

**Authors:** Alexander Vasil’kov, Anastasiia Voronova, Tsvetelina Batsalova, Dzhemal Moten, Alexander Naumkin, Eleonora Shtykova, Vladimir Volkov, Ivanka Teneva, Balik Dzhambazov

**Affiliations:** 1 A.N. Nesmeyanov Institute of Organoelement Compounds, RAS, 119334 Moscow, Russia; voronova.anastasiia.a@mail.ru (A.V.);; 2 Faculty of Biology, Paisii Hilendarski University of Plovdiv, 4000 Plovdiv, Bulgaria; tsvetelina@uni-plovdiv.bg (T.B.); moten@uni-plovdiv.bg (D.M.);; 3 Shubnikov Institute of Crystallography, FSRC “Crystallography and Photonics”, RAS, 119333 Moscow, Russia; eleonora.shtykova@gmail.com (E.S.); vvo@ns.crys.ras.ru (V.V.)

**Keywords:** anticancer drug, gold nanoparticles, iron oxide nanoparticles, drug conjugates, methotrexate, metal-vapor synthesis, X-ray photoelectron spectroscopy, X-ray small-angle scattering

## Abstract

Au and Fe nanoparticles and their conjugates with the drug methotrexate were obtained by an environmentally safe method of metal–vapor synthesis (MVS). The materials were characterized by transmission and scanning electron microscopy (TEM, SEM), X-ray photoelectron spectroscopy (XPS), and small-angle X-ray scattering using synchrotron radiation (SAXS). The use of acetone as an organic reagent in the MVS makes it possible to obtain Au and Fe particles with an average size of 8.3 and 1.8 nm, respectively, which was established by TEM. It was found that Au, both in the NPs and the composite with methotrexate, was in the Au^0^, Au^+^ and Au^3+^ states. The Au 4f spectra for Au-containing systems are very close. The effect of methotrexate was manifested in a slight decrease in the proportion of the Au^0^ state—from 0.81 to 0.76. In the Fe NPs, the main state is the Fe^3+^ state, and the Fe^2+^ state is also present in a small amount. The analysis of samples by SAXS registered highly heterogeneous populations of metal nanoparticles coexisting with a wide proportion of large aggregates, the number of which increased significantly in the presence of methotrexate. For Au conjugates with methotrexate, a very wide asymmetric fraction with sizes up to 60 nm and a maximum of ~4 nm has been registered. In the case of Fe, the main fraction consists of particles with a radius of 4.6 nm. The main fraction consists of aggregates up to 10 nm. The size of the aggregates varies in the range of 20–50 nm. In the presence of methotrexate, the number of aggregates increases. The cytotoxicity and anticancer activity of the obtained nanomaterials were determined by MTT and NR assays. Fe conjugates with methotrexate showed the highest toxicity against the lung adenocarcinoma cell line and Au nanoparticles loaded with methotrexate affected the human colon adenocarcinoma cell line. Both conjugates displayed lysosome-specific toxicity against the A549 cancer cell line after 120 h of culture. The obtained materials may be promising for the creation of improved agents for cancer treatment.

## 1. Introduction

Intensive research on the creation of new drugs and methods to combat oncological diseases continues to be a topical need due to the fact that they are one of the main causes of mortality in the world [1]; almost every sixth death is associated with them. To date, chemotherapy is commonly applied for the treatment of different types of cancer. However, a serious problem that influences chemotherapy effectiveness is the biodistribution of drug compounds, which can cause severe side effects and toxicity [2]. A promising solution in this regard is the development of targeted systems capable of selective delivery of chemotherapeutic agents to cancer cells [3,4,5,6].

For the point transportation of the drug, a suitable marker is needed to identify the cells affected by the neoplastic process. Such a marker may be folic acid receptors, which are known to be overexpressed by cancer cells [7,8,9]. Methotrexate (MTX) is an analogue of folic acid with well-characterized antitumor and anti-inflammatory properties. It is used for the treatment of certain autoimmune diseases (severe psoriasis and rheumatoid arthritis) and different types of cancer (acute lymphoblastic leukemia, osteogenic sarcoma, malignant lymphoma, breast cancer, lung carcinoma, and others) [10,11]. However, significant disadvantages limiting the effectiveness of methotrexate are poor solubility, a short half-life, and rapid diffusion throughout the body [12]. To solve these problems and improve clinical application of the drug, conjugation of methotrexate with nanoparticles was proposed, which showed beneficial effects [13,14,15,16,17,18,19].

Currently, nanoscale materials are gaining more and more popularity due to their effectiveness in targeted drug delivery [20,21,22]. To overcome the traditional disadvantages of chemotherapy, various drug carriers are used: polymer nanoparticles [23], dendrimers [24], polymer micelles [25], and liposomes [26]. In addition, metal nanoparticles (NPs) may represent a new perspective for the development of more effective low-molar-mass chemotherapeutic agents that bypass the multidrug resistance of cancer cells. The properties of metal nanoparticles differ significantly from those of the massive state due to the huge contribution of surface atoms. This leads to a change in the physical, chemical, and biological properties of the materials obtained on their basis. Metal nanoparticles are able to penetrate the cell membrane by endocytosis [27] and can be used for targeted delivery of drugs to cancer cells, minimizing toxicity to normal tissues.

Now, the most studied objects in the field of targeted drug delivery, magnetic resonance imaging, endogenous hyperthermia [28,29,30,31,32,33,34,35,36], biodiagnostic analyses, and photothermal therapy [37,38,39] are gold and iron oxide particles.

Cancer cells have the ability to absorb magnetite nanoparticles by non-specific endocytosis, which means that the use of iron oxide nanoparticles (IONPs) has many advantages, including targeted absorption by cancer cells, modification by various drugs, and the release of heat under the influence of a magnetic field [40]. Coating IONPs with organic compounds to give them specific properties and achieve the ability to bind to specific molecular targets is one of the most promising areas of research [41,42,43,44]. It has been established that iron nanoparticles surface-functionalized with methotrexate are cleaved by intracellular enzymes in lysosomes, which makes it possible to transport methotrexate to the target of cancer cells at low pH, reducing toxicity to normal cells [17].

Hyperthermia is another method of suppressing the growth of cancer cells [45]. To date, the mechanism of cell death has not been studied well enough. The main reason for the ineffectiveness of this method is that it cannot act directly on cancerous tissue. A promising alternative to hyperthermia in the treatment of cancer is the use of heat released by iron nanoparticles under the influence of a magnetic field [46,47]. This allows one to control the amount of medication released by the intensity of the magnetic field, which ultimately increases the effectiveness of chemotherapy.

Gold nanoparticles (Au NPs) exhibit antibacterial and anticancer properties [48,49]. They are inert to human cells, non-cytotoxic, non-immunogenic, and biocompatible [50,51,52]. All these properties indicate that Au NPs as an excellent tool for development of nanocarriers for improved chemotherapy.

Conjugation of chemotherapeutic agents with nanoparticles (NPs) could provide significant benefits for cancer therapy: (1) selective accumulation of the therapeutic agent in cancer tissues, due to the specific properties of nanoparticles; (2) an increase in the antitumor activity of cytostatics due to the manifestation of a synergistic effect when they are in complex with NPs [53,54]. Many developed conjugates of nanoparticles with methotrexate (NPs-MTX) have shown their effectiveness in the treatment of various types of cancer: brain [55,56], colorectal [57], leukemia [58], lung carcinoma [59,60], and breast cancer [61,62]. The introduction of MTX into Au NPs suppressed the growth of lung carcinoma, while an equal free MTX had no effect. Gold-containing nanoparticles modified with methotrexate, when treated with near infrared, demonstrate an anti-inflammatory effect in the treatment of arthritis [63]. Modification of IONPs with methotrexate leads to the minimization of side effects of the drug on normal cells and synchronous therapeutic effects not only due to MTX chemotherapy, but also due to hyperthermia caused by an alternating magnetic field [64].

Metallic NPs can be generated in matrices with various properties, mainly using the chemical formation of metal salts [65,66,67]. However, these methods restrict the biomedical application of the obtained nanomaterials due to the presence of a large number of surfactant impurities, residues of synthetic products, and difficulties controlling the reduction of metals. A convenient and effective alternative to these methods is the metal–vapor synthesis (MVS) [68,69,70], which avoids the use of reducing agents, surfactants, and other unfavorable reagents in the production of monometallic and bimetallic nanoparticles. The synthesis of nanocomposites by MVS is an environmentally friendly and technologically closed cycle. It can be applied for solving one of the priority tasks of pharmacology: the creation of new systems for targeted delivery of antitumor drugs based on conjugates of chemotherapeutic agents with metal nanoparticles. It can be expected that such hybrid materials will show a synergistic effect by enhancing the anticancer activity of the drug due to its conjugation with metal nanoparticles having their own cytotoxic effect. Therefore, the aim of this work was the synthesis of new hybrid materials representing conjugates of methotrexate with metal nanoparticles obtained by MVS, their comprehensive study by physico-chemical methods, and analysis of the possibility of their use as biologically active systems with improved anticancer potential.

## 2. Materials and Methods 

### 2.1. Materials

Acetone (≥99.5%) was dried and distilled over zeolites in an atmosphere of purified argon. Before synthesis, the solvent was degassed by alternating freeze-thaw cycles. Our materials also included metals—gold foil (99.99%); iron foil (99.9%); molybdenum foil (0.25 mm, 99.5%); and tungsten rod (ø 1.5 mm, 99.8%)—and methotrexate (4-Amino-N^10^-methylpteroyl-L-glutamic acid), (Sigma-Aldrich RTC, Inc. 2931 Soldier Springs Rd., Laramie, WY, USA).

### 2.2. Synthesis of Metal Nanoparticles and Their Conjugates with Methotrexate

**Figure 1:**
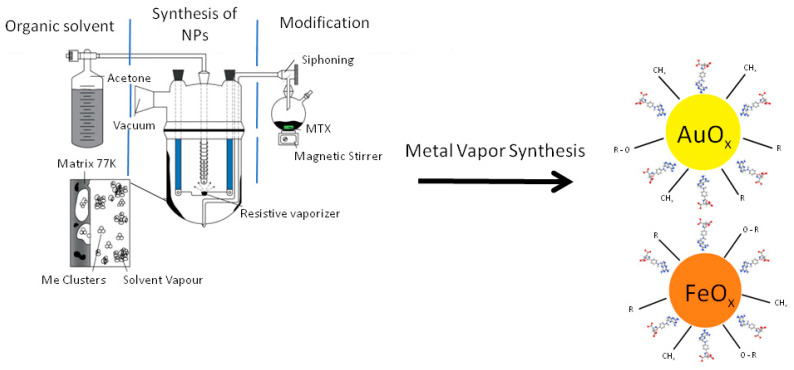
Scheme of synthesis of metal nanoparticles modified with methotrexate.

The preparation of Au and Fe nanoparticles by MVS was described elsewhere [71,72]. In a typical experiment, 0.3 g of metal and 120 mL of acetone were evaporated. Metal vapors were generated in a vacuum of 10^−2^ Pa by the resistive heating of a molybdenum boat for evaporation of Au and a tungsten rod for Fe. Next, joint condensation with acetone took place on the walls of a quartz 5-L reactor cooled by liquid nitrogen (Figure 1). The synthesis was continued for 40–60 min. After the synthesis was completed, the cooling was removed, and the co-condensate matrix was heated to room temperature. Organosols of metals in situ were siphoned from the reactor into two evacuated flasks. Several typical experiments were carried out. In the first series, organosols of Au and Fe in acetone were obtained which were, in situ, siphoned from the reactor into evacuated flasks. Acetone was distilled from them, and the resulting metal billets were examined. In the second series, synthesized organosols of metals were siphoned from the reactor into evacuated flasks containing prepared MTX (30 mg) solutions in acetone. Modification was carried out in an argon atmosphere at a temperature of 40 °C for 20 min with intensive stirring by a magnetic stirrer. After modification, the acetone was distilled, and the resulting conjugates were examined.

### 2.3. Morphology of the Obtained Samples

The surface morphology of the nanocomposites was studied with scanning electron microscopy (SEM) using a Hitachi TM4000Plus microscope (Tokyo, Japan) at an accelerating voltage of 15 kV in the secondary electron mode. Energy dispersive X-ray studies (EDX) were carried out using a QUANTAS 75 spectrometer, Bruker (Billerica, MA, USA).

Metal nanoparticles were analyzed with transmission electron microscopy (TEM) using the LEO 912AB OMEGA device (Zeiss, Jena, Germany) at an accelerating voltage of 100 kV. For analysis, the samples were pre-crushed in an agate mortar, suspended in ethanol and dispersed by ultrasound for 10–15 min. Next, a small amount was placed on a formvar/carbon mesh. The particle size distribution of the samples was calculated by measuring the size of 200 displayed particles using the SigmaScan Pro 5.0 software.

### 2.4. Thermogravimetric Analysis

Thermogravimetric analysis was carried out using a Derivatograph-C device (MOM, Mátészalka, Hungary) on samples weighing ~15 mg at a heating rate of 5 °C/min in an argon atmosphere.

### 2.5. X-ray Photoelectron Spectroscopy

X-ray photoelectron spectra were obtained using an Axis Ultra DLD (Kratos Ltd., Manchester, UK) spectrometer using monochromatic Al Kα radiation at an X-ray gun power of 150 W. Survey and high-resolution spectra were recorded at pass energies of 160 and 40 eV and with step sizes of 1 and 0.1 eV, respectively. The size of the analyzed area was about 300 μm × 700 μm. The samples were fixed on a holder using a double-sided adhesive tape and examined at room temperature at a residual pressure in the spectrometer chamber not exceeding 10^−8^ Torr. The pre-calibration of the spectrometer energy scale corresponded to the following peak value of the standard sample (the metal surface purified by ion sputtering): Au 4f_7/2_–83.96 eV. To eliminate the effect of charging the samples, the spectra were taken using a neutralizer. The spectra were referenced to the C-C/C-H state isolated in the C 1s spectrum, to which energy of 285.0 eV was attributed. The background of inelastic electron energy losses was subtracted using the Shirley method.

### 2.6. Small-Angle X-ray Scattering

SAXS measurements were taken on a laboratory diffractometer, “AMUR-K” (Institute of Crystallography, Moscow, Russia), at a wavelength of *л* = 0.1542 nm in a Kratky-type (infinitely long slit) geometry covering the range of momentum transfer 0.12 < *s* < 7.0 nm^−1^ (here, *s* = 4*p sin и*/*л*, where 2*θ* is the scattering angle). The scattering profiles were corrected for background scattering and primarily processed using the program PRIMUS [73] of the software suite ATSAS [74]. The experimental SAXS data were normalized for the intensity of the incident beam, and then a correction for the collimation distortion was made in accordance with the standard procedure [75].

The processed experimental SAXS curves were used to compute the volume size distribution functions *D_V_*(*R*) of the scattering particles. Assuming the particles to be spherical, an indirect transform program GNOM [76] was employed to solve the integral equation

(1) $$I(s)\;=\;{\left(\Delta \rho \right)}^{2}\int_{R_{\min}}^{R_{\max}}D_{V}(R)\;m^{2}(R)\;i_{0}(sR)\;dR,$$

where *R* is the radius of a sphere, *i*_0_(*x*) = {[*sin* (*x*) − *x cos* (*x*)]/*x*^3^}^2^ is the sphere form factor, and *m*(*R*) = (4*p*/3)*R*^3^Δ*ρ*, where Δ*ρ* is the particle contrast. The value of *R_min_* was kept zero; that of *R_max_* was selected for each individual data set by successive runs with different values of this parameter to obtain values that fit the experimental data.

### 2.7. Powder X-ray Diffraction

Powder X-ray diffraction (PXRD) phase analysis was performed with a D8 Advance (Bruker AXS, Karlsruhe, Germany) diffractometer in the Bragg-Brentano focusing geometry using CuK_α_ radiation and an angular range of 5–90° with a step of 0.02° and the scan rate of 0.5–2 deg min^−1^. The samples were placed on flat holders. Diffraction pattern profiles were fitted using the TOPAS 5 program package (Bruker AXS).

### 2.8. Cell Types and Culture Conditions

The cytotoxicity and potential antitumor effects of Au NPs, Fe NPs, and their conjugates were analyzed in vitro using four human cell lines: A549 (American Type Culture Collection /ATCC/ number CCL-185™) derived from human lung adenocarcinoma; HeLa (ATCC CCL-2™) with human cervical adenocarcinomic origin; HT-29 (ATCC HTB-38 HT29) derived from colorectal adenocarcinoma; and HFL1—human fetal lung fibroblasts (CLS Cell Lines Service GmbH, Eppelheim, Germany). The HFL1 cells served as a noncancerous control. All cancer cell lines were cultured in Dulbecco’s modified Eagle’s medium (DMEM) (Merck KGaA, Darmstadt, Germany) supplemented with 10% fetal calf serum (FCS) (Merck KGaA, Darmstadt, Germany), 100 μg/mL streptomycin, and 100 IU penicillin (Merck KGaA, Darmstadt, Germany). HFL1 cells were grown in DMEM containing 15% FCS and the same amount of antibiotics. Antifungal agents were not added to the cell culture medium with the aim of avoiding negative effects on the cells. It has been demonstrated that the commonly used amphotericin B intercalates and affects the functions of both fungal and mammalian cell membranes [77]. All cell cultures were routinely monitored for biological contamination. The cells were grown under standard conditions: 37 °C, 5% CO_2_-95% atmospheric air, high humidity, and expanded up to 80% confluency in 75 cm^2^ culture flasks (TPP, Trasadingen, Switzerland) prior to all experiments.

### 2.9. In Vitro Cytotoxicity Assays

In vitro assays based on reduction of 3-(4,5-dimethylthiazol-2-yl)-2,4-diphenyl tetrazolium bromide (MTT) and neutral red (NR) uptake were performed to evaluate the cytotoxic and anticancer potential of NPs and NPs-MTX conjugates. All cell types were harvested at 70–80% confluency. Cell suspensions with a concentration of 1 × 10^5^ cells/mL were seeded (100 μL/well) on 96-well plates (TPP, Trasadingen, Switzerland) and cultured for 24 h. After that, the cells were treated with different concentrations of NPs and NPs-MTX. The nanoparticle samples were suspended in Dulbecco’s phosphate buffered saline (DPBS) (Merck KGaA, Darmstadt, Germany) in a concentration of 5 mg/mL and sonicated for 300 s. The samples were tested in four different concentrations: 10, 50, 100, and 200 μg/mL. For this aim, NP stock solutions were diluted in cell culture medium. The cells were incubated with NPs for 24, 48, 72, 96, and 120 h. Methotrexate served as a positive control for all in vitro cytotoxicity tests and was assayed in the same concentrations as the nanoparticles and NPs-MTX samples. An equivalent volume of culture medium was added to the control cells that served as untreated control. At the end of every test period MTT (Merck KGaA, Darmstadt, Germany) or NR (Merck KGaA, Darmstadt, Germany) solution was added to the cells, achieving a final concentration of 0.5 mg/mL. The culture plates were incubated for 2–4 h in the dark at 37 °C, 5% CO_2,_ and high humidity. Then, the culture medium was discarded and the cells were washed with D-PBS (Merck KGaA, Darmstadt, Germany). The formazan accumulated in cells was solubilized with dimethyl sulfoxide (100 μL/well DMSO) for 15 min under continuous mild shaking. After that, absorbance at 570 nm wavelength was determined using a Synergy-2 reader (BioTek, Winooski, VT, USA). Respectively, for NR assays after washing with D-PBS, the NR dye accumulated in cells was extracted with 100 μL/well solution containing 50% ethanol and 1% acetic acid. The cells were incubated for 15 min under continuous mild shaking followed by measurement of absorption at 540 nm using a Synergy-2 reader (BioTek, Winooski, VT, USA). All assays were performed with triplicate samples. Results were displayed as percent inhibition of cell viability and metabolic activity which was calculated using the data from treated cells and cells cultured in standard conditions without test-sample as previously described [78]. IC_50_ values were calculated based on MTT assay results.

### 2.10. Spheroid Tests

HT29 cells were detached at 70–80% culture confluency, and 5000 cells/well in a culture medium volume of 200 μL were seeded on 96-well clear round bottom spheroid microplates (Corning Inc., Glendale, AZ, USA). After 24 h the formed 3D spheroids were treated with 100 μg/mL NPs, NPs-MTX, or MTX for a total period of 96 h. Spheroids cultured for the same period in normal growth medium served as untreated control. Measurements of spheroid diameter after 24, 72, and 96 h cultured were performed on an Inverso microscope (Medline Scientific, Chalgrove, Oxon, UK) using a high definition digital camera Si-3000 and software (Medline Scientific, Chalgrove, Oxon, UK).

## 3. Results

### 3.1. Morphology of the Samples

In real systems, the properties of materials based on metal nanoparticles are determined by the composition and size of the particles and the nature of their specific interaction with the stabilizing carrier, as well as the conditions for obtaining the material.

**Figure 2:**
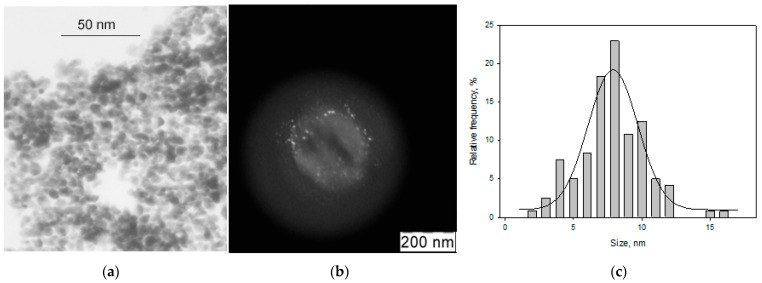
TEM images in a light (**a**) and dark (**b**) field, and the size distribution (**c**) of Au nanoparticles in the Au-acetone system.

Figure 2 shows TEM micrographs in a light (a) and dark (b) field, and a histogram of the size distribution of Au nanoparticles from the Au-acetone system (AuAc).

Analysis of the micrographs showed that the particles have a spherical shape with an average size of 8.3 nm.

**Figure 3:**
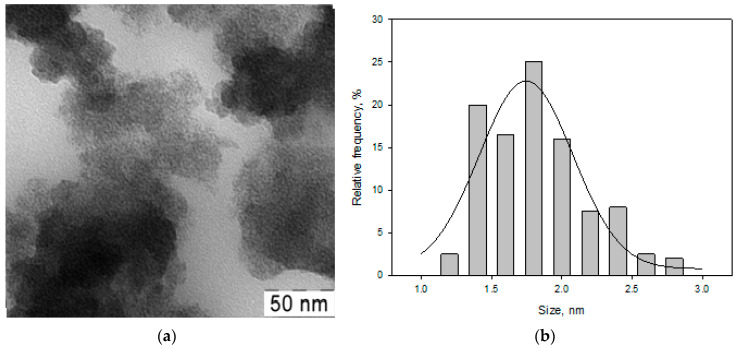
TEM images (**a**) and the size distribution (**b**) of Fe nanoparticles from the Fe-acetone system.

Figure 3 shows TEM micrographs (a) and the size distribution (b) of Fe nanoparticles from the Fe-acetone (FeAc) system.

Small particles with an average size of 1.8 nm and a blurred profile were recorded, which may indicate a loose particle surface, apparently associated with oxidation.

**Figure 4:**
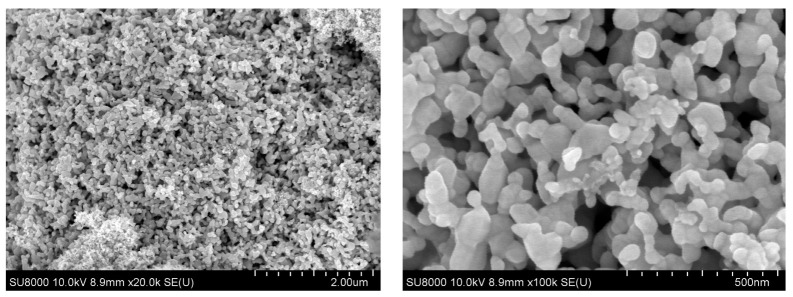
Au NPs SEM micrographs at various magnifications.

Analysis of the morphology of Au nanoparticles obtained after isopropanol removal was carried out using the SEM method. It has been established that metal particles combine into aggregates, which are aggregates of smaller nanoparticles. The Au NPs micrograph is shown in Figure 4.

**Figure 5:**
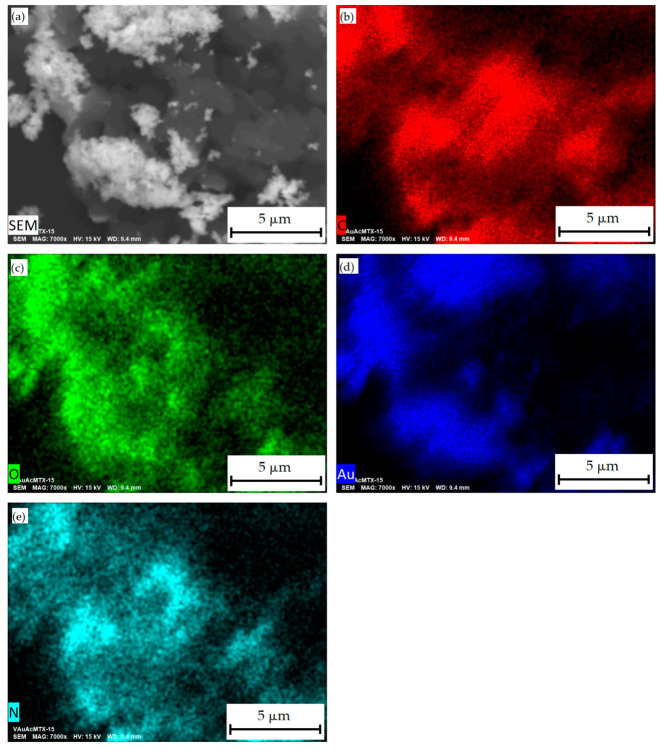
SEM-image of morphology of AuAcMTX conjugate nanoparticles (**a**) and its elemental mappings: C (**b**), O (**c**), Au (**d**), and N (**e**).

The SEM gave an idea of the morphology of the surface of the samples modified by MTS. Gold and methotrexate nanoparticles are evenly distributed (Figure 5). It was found that conjugates of Au nanoparticles from the Au-acetone system with methotrexate (AuAcMTX) after removal of the dispersion medium form poly-dispersed aggregates with a wide particle size distribution. Mapping showed homogeneous distributions of Au and N; the latter is an indicator of the presence of methotrexate.

**Figure 6:**
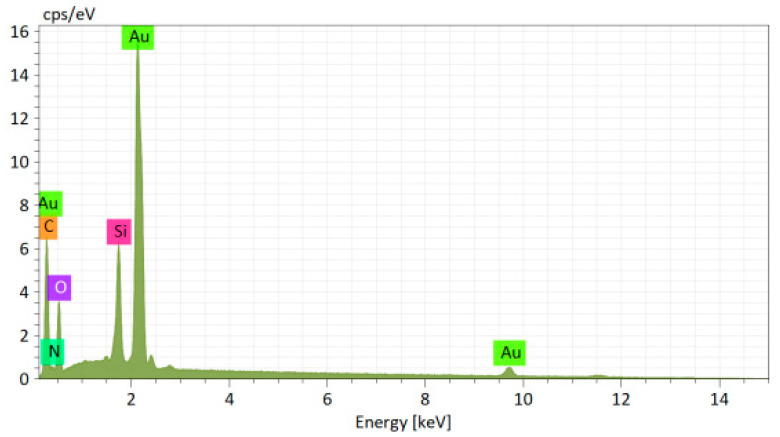
Energy dispersive X-ray spectrum from AuAcMTX: C—58.5 at. %; O—20.1 at. %; N—7.0 at. %; Au—8.4 at. %.

The elemental composition of the AuAcMTX conjugate is presented in the EDX spectrum (Figure 6).

**Figure 7:**
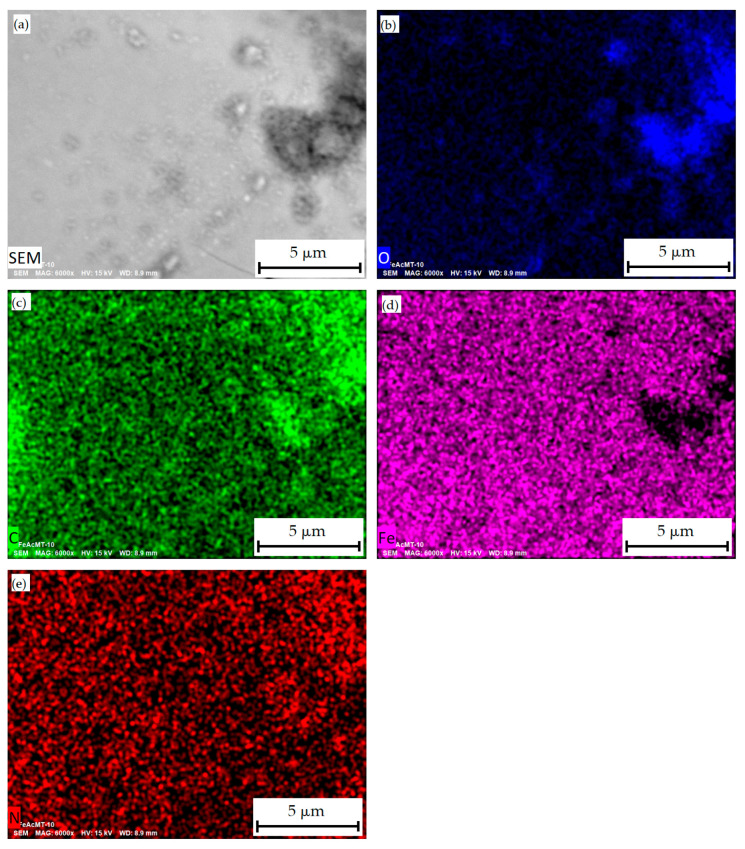
SEM-image of morphology of FeAcMTX conjugate nanoparticles (**a**) and its elemental mappings: C (**b**), O (**c**), Fe (**d**), and N (**e**).

Analysis of SEM micrographs for Fe nanoparticles from the Fe-acetone system modified by MTX (FeAcMTX) showed that Fe and N nanoparticles are distributed evenly throughout the sample volume, with the latter serving as an indicator of the presence of methotrexate (Figure 7).

**Figure 8:**
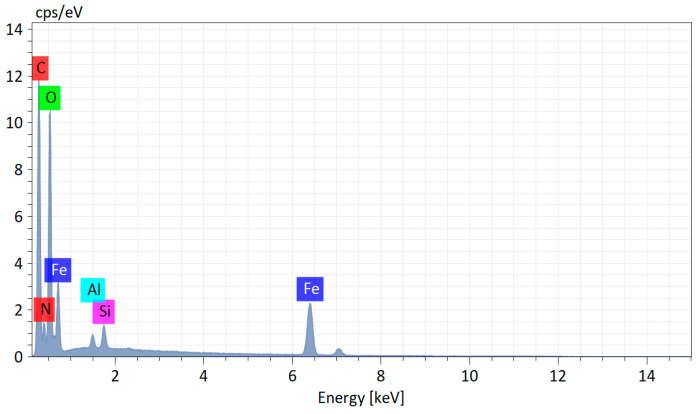
Energy dispersive X-ray spectrum from FeAcMTX: C—51.5 at. %; O—37.3 at. %; N—12.9 at. %; Fe—14.1 at. %.

The elemental composition of the FeAcMTX conjugate is presented in the EDX spectrum (Figure 8).

### 3.2. Thermogravimetric Analysis

Samples of pure methotrexate and methotrexate modified with metal nanoparticles were examined using thermogravimetric analysis. Figures S1–S3 show thermograms of metal nanoparticles obtained from organosols with acetone.

Analysis of the TGA and DTA curves shows that methotrexate and Fe nanoparticles (Figures S1 and S2) are characterized by low-temperature mass loss due to the removal of the sorbed solvent, unlike the Au sample. Methotrexate melts degraded at temperatures above 230 °C. Fe oxidation began at a temperature of 350 °C. For Au samples, the mass in the air does not change, while a 2% mass loss is observed in argon (Figure S3a).

For methotrexate conjugates with iron, there are no changes in the TGA curve compared to the NPs curve, however, DTA demonstrates a sharp exo-effect in the region of 200 °C and 300 °C, which cannot be associated with Fe oxidation (Figure S2b). The exo-effects of methotrexate conjugate with Au in air and pure NPs in air are absolutely different (Figure S3b). It can be assumed that this behavior is due to the chemical interaction of metal nanoparticles with methotrexate.

### 3.3. X-ray Photoelectron Spectroscopy

**Figure 9:**
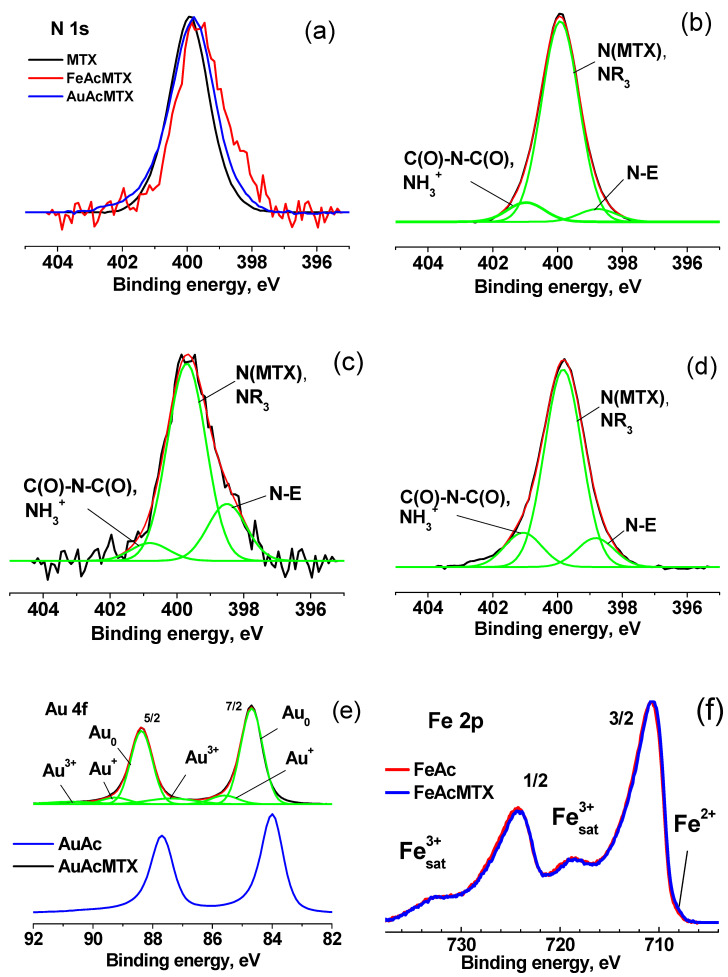
Photoelectron spectra of MTX, FeAcMTX and AuAcMTX: N 1s spectra of MTX, FeAcMTX, and AuAcMTX, normalized by intensity of main peak (**a**); N 1s spectra of MTX (**b**), its conjugates with Fe (FeAcMTX) (**c**) and Au (AuAcMTX) (**d**); Au 4f photoelectron spectra of AuAc AuAcMTX (**e**); Fe 2p spectra of FeAc and FeAcMTX normalized by the intensity of the main peak (**f**).

The surface of nanoparticles is one of the important components determining the physico-chemical characteristics of nanoparticles; therefore, FeAc, AuAc samples, and their conjugates FeAcMTX and AuAcMTX were studied using surface-sensitive XPS. Photoelectron spectroscopy is the leading analytical method for the characterization of various chemical states of elements on the surface. Earlier, studies of the electronic state of gold and iron in conjugates with methotrexate synthesized by other methods were carried out [16,79]. In the survey spectra of the samples shown in Figures S4–S8, along with the peaks corresponding to metals, there are peaks from the elements that make up the solvent and methotrexate. Figure 9a–d shows the N 1s photoelectron spectra of methotrexate and its conjugates with Fe and Au nanoparticles, approximated with three Gaussian peaks, the characteristics of which are given in Table 1.

The peak characteristic of methotrexate has a binding energy of about 399.9 eV, which varies slightly from sample to sample. The binding energy of about 398.8 eV is not characteristic of C-N bonds [80]. It can be caused by the electronic effect of intermolecular interactions and, as a rule, corresponds to the bonds of nitrogen atoms with metals. This N-E state is registered in a small amount in methotrexate, but increases significantly in its conjugates with metals. After the interaction of MTX with Fe nanoparticles, the relative intensity of this peak increases by about 3 times and with Au nanoparticles by 1.5 times. The binding energy of about 401 eV is intermediate between the values of 400.6 and 401.46 eV, characteristic of the C(O)-N-C(O) and NH^3+^ groups and, apparently, can be attributed to intermolecular interactions with the formation of hydrogen bonds between oxygen atoms of the C(O)N groups and hydrogen atoms methotrexate molecules. The relative intensity of this component in AuAcMTX is 1.6 more than that of MTX.

Figure 9e shows the Au 4f photoelectron spectra of Au NPs and its conjugate with methotrexate. The Au 4f_7/2_ peak in the spectrum of AuAc nanoparticles at 84.7 eV could be attributed to the Au^+^ state provided that a peak width is much larger (~0.9 eV). However, in the case under consideration, its width practically coincides with that of the AuAcMTX sample. Therefore, it can be concluded that Au nanoparticles are surrounded by an organic shell that prevents charge drain. After applying methotrexate, the spectrum shifts by −0.7 eV towards lower binding energies, which indicates the appearance of a channel for charge drain caused by the transformation of the hydrocarbon shell and the elimination of differential charging, and the presence of Au^+^ and Au^3+^ states is observed (peaks at 85.6 and 87.4 eV). The Au 4f spectra for Au-containing systems are very close. The effect of methotrexate is manifested in a slight decrease in the proportion of the Au^0^ state—from 0.81 to 0.76.

Figure 9f demonstrates the coincidence of the Fe 2p spectra of the FeAc and FeAcMTX samples. This may indicate that methotrexate has a weak effect on the Fe^3+^ state characteristic of Fe nanoparticles. The Fe^2+^ state is present in an insignificant amount. The modification of nanoparticles with methotrexate leads to a partial reduction and a slight increase in its concentration.

### 3.4. Powder X-ray Diffraction

**Figure 10:**
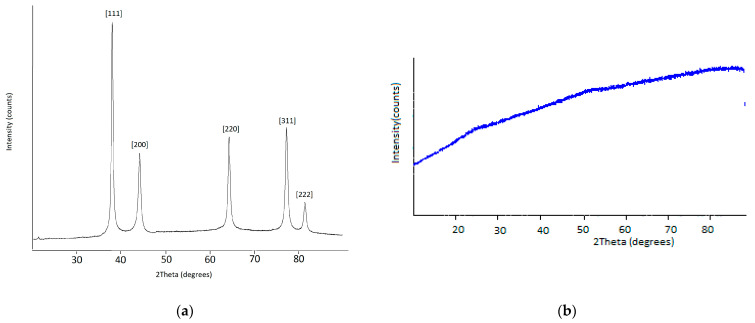
PXRD diffractograms of Au NPs (**a**) and FeO_x_ NPs (**b**).

The powdered Au and FeO_x_ NPs obtained using the MVS were studied with the powder X-ray diffraction. Figure 10 shows the PXRD pattern of the Au and FeO_x_ powders prepared acetone as an organic reagent. The corresponding peaks are clearly seen in Figure 10a at the 2θ angle values of 38.1° [111], 44.2° [200], 64.4° [220], 77.4° [311], and 81.3° [222]. The powder XRD pattern is characteristic of the face-centered-cubic phase of Au^0^ (JCPDS No: 04-0784), and the crystallite size calculated using the Scherrer equation is 12.3 nm. The powder XRD pattern of FeOx NPs (Figure 10b) shows that the sample is not crystalline but is an amorphous material. The PXRD shows no differences between the diffractograms of metal nanoparticles and their conjugates with methotrexate.

### 3.5. Small-Angle X-ray Scattering

To analyze the size distribution of Fe NPs and Au NPs obtained in an acetone medium and their conjugates with methotrexate, small-angle X-ray scattering (SAXS) was used, which makes it possible to determine the structure of the material in the range of 1–250 nm.

**Figure 11:**
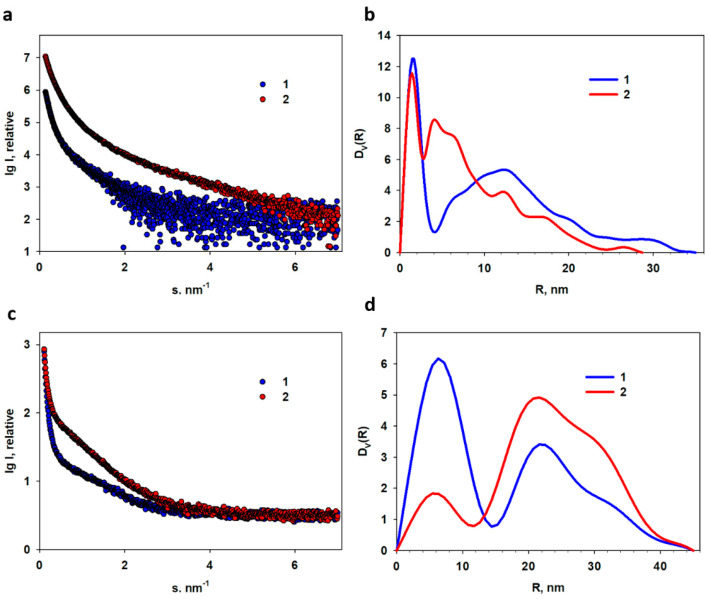
SAXS patterns from the iron and gold nanoparticles obtained in an acetone medium and their conjugates with methotrexate: (**a**) experimental SAXS curves from AuAc (1) and AuAcMTX (2) nanoparticles; (**c**) experimental SAXS curves from FeAc and FeAcMTX nanoparticles; (**b**,**d**) the volume size distribution functions *D_V_*(*R*) calculated from the curves shown in the panels (**a**,**c**).

The experimental scattering profiles from Fe NPs and Au NPs obtained in an acetone medium along with those from their conjugates with methotrexate are presented in Figure 11a,c.

All profiles of the experimental SAXS curves are characteristic of polydisperse compounds. The initial portions of the SAXS patterns close to the primary beam (scattering vectors less than s ≈ 1.0 nm^−1^) display a strong upward trend, indicating that a significant portion of large aggregates is present in each of the samples. Moreover, scattering by spherical nanoparticles (form factor) is practically absent in the experimental curves.

Only a weak presence of a form-factor, possibly from the individual particles and/or their small aggregates, is observed in the SAXS curves from the pure iron nanoparticles and those with methotrexate (Figure 11c). The high polydispersity of the samples and possible interaction between metal nanoparticles limits the structural task by searching for the size distribution in the spherical approximation of scattering inhomogeneities [75].

The distribution functions *D_V_*(*R*) obtained by this method for all the studied samples are shown in Figure 11b,d. All functions are generally bimodal. It is important that, for all samples, SAXS reveals highly homogeneous populations of the individual NPs coexisting with a wide fraction of large aggregates, the number of which increases significantly in the presence of methotrexate. The narrowest distribution with the smallest individual nanoparticles (*R_small_* = 1.5 nm) belongs to Au NPs obtained in acetone (Figure 11b). Large aggregates of this sample reach a size with *R_max_* = 35 nm. The fraction of these particles has a wide profile with a maximum for the particles with radius equal to 12.5 nm, what is comparable with those synthesized using HAuCl_4_ and water-soluble chitosan solution [81]. For conjugates of Au with methotrexate, the *D_V_*(*R*) distribution profile has changed: a new fraction of particles with radii of approximately 4 and 6 nm was formed, and the maximum radius slightly decreased to 30 nm. It can be assumed that particles with a radius of 4 and 6 nm are small clusters consisting of 2 and 3 individual gold nanoparticles connected by methotrexate. It should be noted that methotrexate, being a low molar mass substance that does not contain heavy atoms, does not contribute to the scattering. Apparently, methotrexate most likely interacts with hydrocarbon shells on the surface of the metal nanoparticles, forming aggregates or clusters of various sizes and changing the whole pattern of the scattering. The widest fraction of individual metal nanoparticles is observed for the iron nanoparticles with *R_small_* ≈ 6.0 nm. The radius of aggregates varies from 20–23 nm (maximum of size distribution function in this region) up to 45 nm. In the presence of methotrexate, the number of aggregates greatly increases, while the fraction of individual iron nanoparticles sharply decreases. At the same time, no new fractions are formed and the main dimensions remain the same. It was found that for all samples, the SAXS registers highly heterogeneous populations of nanoparticles coexisting with a wide proportion of large aggregates, the number of which increases significantly in the presence of methotrexate. Methotrexate, being a low-molecular compound, practically does not contribute to small-angle scattering, but leads to an intensification of interparticle interactions. This may indirectly indicate the presence of the chemical interaction of metal nanoparticles with methotrexate and the formation of hybrid forms with significantly larger sizes than the original metal nanoparticles.

### 3.6. Biological Evaluations of Nanoparticles and Their Conjugates with Methotrexate

**Figure 12:**
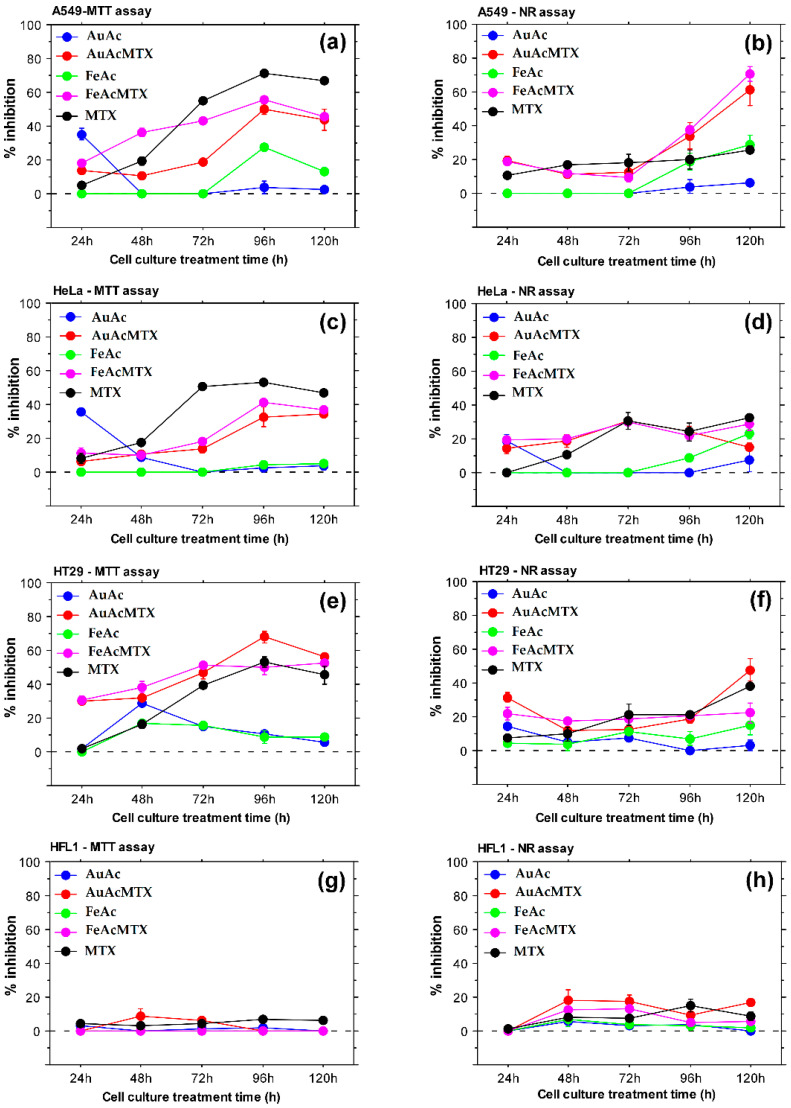
Longitudinal evaluation of metal nanoparticles and NPs-MTX conjugates in vitro cytotoxicity. (**a**,**c**,**e**,**g**) show the inhibition of cellular metabolic activity determined by MTT assay with A549 (**a**), HeLa (**c**), HT29 (**e**), and HFL1 (**g**) cells treated with 100 μg/mL test-compound. (**b**,**d**,**f**,**h**) show NR assay results for A549, HeLa, HT29, and HFL1 cells, respectively, cultured in medium containing 100 μg/mL AuAc, FeAc, AuAcMTX, FeAcMTX, and MTX for 24, 48, 72, 96, and 120 h. All samples were analyzed in triplicate.

In vitro assays with human cell lines demonstrated concentration- and time-dependent responses towards treatment with nanoparticles and NPs-MTX conjugates (Table 2; Figure 12). Overall, AuAc and FeAc showed a good biocompatibility profile and induced mild cytotoxic effects after extended treatment periods (96 and 120 h) selectively against cancer cells (Figure 12a,b,d,f). These results point to an anticancer effect because the noncancer HFL1 cells showed low levels of metabolic activity and viability inhibition compared to the cancer cell lines (Figure 12g,h).

MTT assay results demonstrated stronger cytotoxicity of conjugates with methotrexate compared to methotrexate alone against HT29 cells (Figure 12e). NR uptake data also showed (Figure 12b) higher activity of NPs loaded with methotrexate confirming effective drug-NPs conjugation.

**Figure 13:**
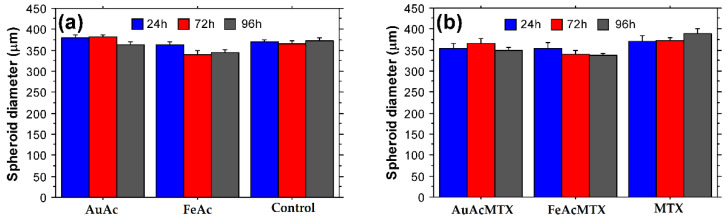
Effects of AuAc, FeAc, AuAcMTX, FeAcMTX, and MTX on HT29 spheroid size. HT29 spheroids were treated with 100 μg/mL AuAc, FeAc, AuAcMTX, FeAcMTX, or MTX for 96 h and the diameter of multicellular aggregates was measured at different time-points (24, 72, and 96 h). (**a**) Spheroid size following treatment with AuAc or FeAc. Control—spheroids cultured in standard growth medium without addition of NP samples. (**b**) HT29 spheroid dimensions after treatment with AuAcMTX, FeAcMTX, or MTX. All samples were assayed in triplicate.

AuAcMTX specifically affected human colon adenocarcinoma cell line HT-29 to the highest extent. FeAcMTX showed the highest toxicity against lung adenocarcinoma cells A549. Both samples displayed higher lysosome-specific toxicity against the A549 cancer cell line after 96 and 120 h of culture as evident from the results of the NR assay. The detected increased toxicity of AuAcMTX and FeAcMTX against HT29 cells was also confirmed with tumor spheroid assays (Figure 13). A reduction in spheroid size was detected for both FeAc and FeAcMTX conjugates while treatment with MTX did not lead to similar effects on 3D tumor structures. Incubation with MTX alone did not influence HT29 spheroid size, which suggests synergistic effects of samples loaded with methotrexate on the level of multicellular tumor structure.

The present study demonstrates the production of biocompatible gold and iron nanoparticles with potential to serve as effective drug carriers. Standard in vitro assays were used to evaluate the cytotoxicity of the synthesized conjugates. The MTT assay provides a valuable tool for estimation of mitochondrial metabolic activity and cellular viability following treatment with NPs and NP-MTX conjugates [82]. The NR uptake assay is used for the evaluation of lysosome functionality and cell culture viability in response to treatment with a test agent [83]. The two assays, applied on a panel of cell lines including both cancer and noncancerous cells, allow estimation of general cytotoxic effects, organelle-specific toxicity, and anticancer potential. When combined with 3D spheroid analyses, the results convincingly demonstrate antitumor effects that could be achieved on a cancer-tissue-specific level. Indeed, we observed a reduction in tumor spheroid size following treatment with iron oxide NPs and conjugates with methotrexate (Figure 12a,b). These results together with the detected time-dependent lysosome-specific cytotoxicity of AuAcMTX and FeAcMTX point to a synergistic effect of the nanoparticles in combination with the chemotherapeutic agent.

The effectiveness of drug-loaded gold and iron nanocarriers against various cancer cell types was demonstrated in several reports [15,17,82,84]. Particularly, our results confirm the recent data [16] that showed inhibitory effects of Au NPs-MTX against lung and colon cancer cells. They also detected stronger cytotoxicity levels in colon cancer cell line HTC-116 and documented proapoptotic activity of the tested NP samples. Our results supplement these findings by indicating a cancer-specific cytotoxicity of NPs-MTX conjugates with pronounced effects on lysosome functionality in lung cancer cells. The effect was detected after an extended treatment period (96 h, 120 h). In addition, a reduction in size of HT29 tumor spheroids cultured in the presence of NPs-MTX suggests cancer-specific toxicity on a multicellular level and improved cancer-tissue-specific effectiveness.

## 4. Conclusions

New biologically active materials, which are conjugates of methotrexate with Au and Fe nanoparticles, were obtained using an environmentally safe method of metal–vapor synthesis. Au and Fe particles with sizes of 8.3 and 1.8 nm, respectively, were synthesized by the interaction of metal vapors and acetone as a dispersion medium. The surface analysis by XPS of both metal nanoparticles and their conjugates with methotrexate showed that Au both in the Au NPs and the composite with methotrexate are in Au^0^, Au^+^, and Au^3+^ states. In the Au 4f_7/2_ spectra of Au nanoparticles and their conjugate, a change in the binding energy of the metal from 84.7 to 84.0 eV was recorded, which is accompanied with a decrease in the fraction of the Au^0^ state from 0.81 to 0.76. In the Fe conjugates, the main state is the Fe^3+^ state, while the Fe^2+^ state is present in a small amount. Modification of Fe nanoparticles with methotrexate leads to a partial reduction in the metal. Analysis of the N 1s spectra of the conjugates showed an increase in the intensity of components at ~398.8 eV, which may be attributed to nitrogen–metal (N–M) interactions. When interacting with Fe nanoparticles, the relative intensity of this peak increases by 3 times, whereas for Au it is about 1.5 times.

Small-angle X-ray scattering was applied to analyze the dimensions of both Fe and Au nanoparticles and their conjugates with methotrexate. For all samples, SAXS reveals highly homogeneous populations of the individual NPs coexisting with a fraction of large aggregates, the number of which increases significantly in the presence of methotrexate. Being a low molar mass compound, methotrexate practically does not contribute to small-angle scattering, but leads to intensification of interparticle interactions and, as a result, to the aggregation of metal nanoparticles.

Biological evaluations of the generated nanomaterials indicated low cytotoxic effects of monometallic Au and Fe NPs against cancer cell lines, which increased specifically for A549 and HeLa cells following extended culture periods (96 h, 120 h). AuAcMTX conjugates affected the human colon adenocarcinoma cell line HT-29 to the highest extent. FeAcMTX showed the highest toxicity against lung adenocarcinoma cells. Both samples (AuAcMTX and FeAcMTX) induced lysosome-specific toxicity in A549 cells after treatment for more than 72 h and reduced the size of HT29 tumor spheroids. The present study indicates a potential for increased activity of methotrexate against colon and lung adenocarcinoma cells based on conjugation of the drug with Au and Fe NPs and a possible drug-NPs time-dependent synergistic effect. Therefore, the generated MTX-NPs conjugates could represent new tools for improvement of chemotherapy effectiveness.

## Figures and Tables

**Table 1:** Parameters of components in the N 1s photoelectron spectra of the methotrexate and its conjugates with metal nanoparticles: E_b_—binding energy, W—peak width, and I_rel_—relative intensity.

Example	Group	C(O)-N-C(O), NH^3+^	N (MTX), NR_3_	N-E
MTX	E_b,_ eV	401.0	399.9	398.8
	W, eV	1.15	1.15	1.15
	I_rel_, %	0.083	0.862	0.055
FeAcMTX	E_b,_ eV	400.8	399.8	398.6
	W, eV	1.15	1.15	1.15
	I_rel_, %	0.064	0.714	0.222
AuAcMTX	E_b,_ eV	401.1	399.8	398.8
	W, eV	1.15	1.15	1.15
	I_rel_, %	0.132	0.757	0.111

**Table 2:** IC_50_ values (μg/mL) determined by MTT assays following 72 h treatment with test-compounds.

Samples	A549	HeLa	HT29	HFL1
AuAc	n.d.	n.d.	n.d.	n.d.
FeAc	n.d.	n.d.	n.d.	n.d.
MTX	47.63 (±2.15)	86.82 (±13.18)	n.d.	n.d.
AuAcMTX	n.d.	163.64 (±1.84)	110.48 (±6.85)	n.d.
FeAcMTX	n.d.	n.d.	95.64 (±2.13)	n.d.

n.d.—not determined.

## Data Availability

Data will be available upon reasonable request from the corresponding author.

## Supplementary Materials

The following supporting information can be downloaded at: https://www.mdpi.com/article/10.3390/ma16083238/s1, Figure S1: TGA and DTA curves for MTX in air and argon; Figure S2: TGA and DTA curves in air and argon: (a) for FaAc; (b) for FeAcMTX; Figure S3: TGA and DTA curves in air and argon: (a) for AuAc; (b) for AuAcMTX; Figure S4: Survey XPS spectra of the MTX sample; Figure S5: Survey XPS spectra of the AuAc sample; Figure S6: Survey XPS spectra of the AuAcMTX sample; Figure S7: Survey XPS spectra of the FeAc sample; Figure S8: Survey XPS spectra of the FeAcMTX sample.
