# Supplementary material for: Evolution of Gold and Iron Oxide Nanoparticles in Conjugates with Methotrexate: Synthesis and Anticancer Effects
Source: Materials (Basel). 2023 Apr 19;16(8):3238. doi: 10.3390/ma16083238 (PMC10146258; doi:10.3390/ma16083238)
Supplement: Supplementary file 1 [file materials-16-03238-s001.zip › materials-2305270-supplementary.pdf]

Supplementary Materials

Evolution of Gold and Iron Oxide Nanoparticles in Conjugates with Methotrexate: Synthesis and Anticancer Effects

Alexander Vasil'kov [1,*], Anastasiia Voronova [1], Tsvetelina Batsalova [2], Dzhemal Moten [2], Alexander Naumkin [1], Eleonora Shtykova [3], Vladimir Volkov [3], Ivanka Teneva [2] and Balik Dzhambazov [2]

[1] A.N. Nesmeyanov Institute of Organoelement Compounds, RAS, 119334 Moscow, Russia
[2] Faculty of Biology, Paisii Hilendarski University of Plovdiv, 4000 Plovdiv, Bulgaria
[3] Shubnikov Institute of Crystallography, FSRC "Crystallography and Photonics", RAS, 119333 Moscow, Russia
* Correspondence: alexandervasilkov@yandex.ru

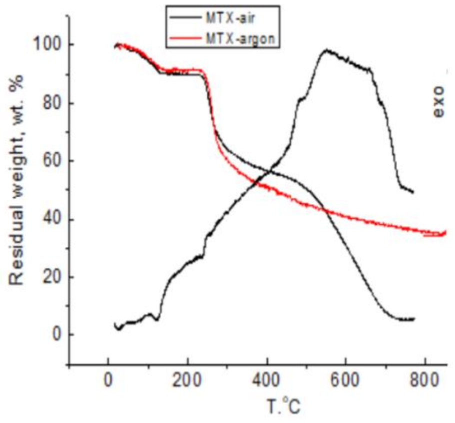

Figure S1. TGA and DTA curves for MTX in air and argon.

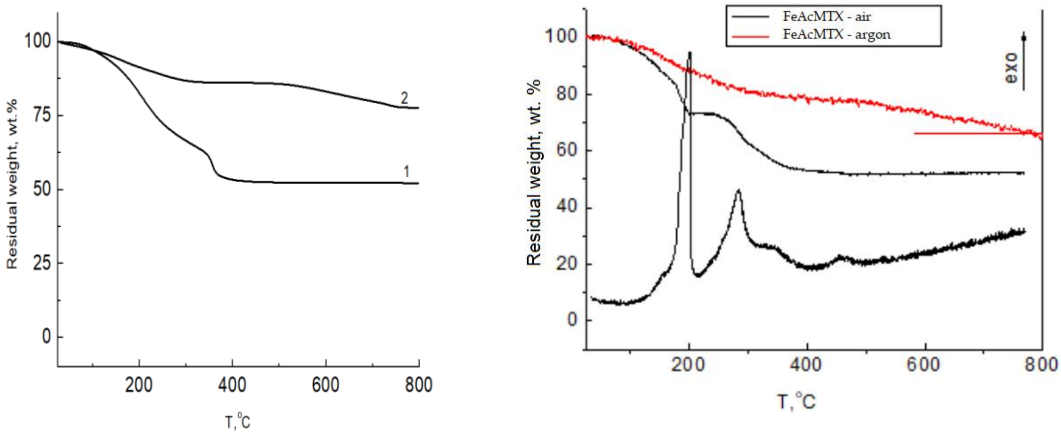

(**a**) (**b**)

Figure S2. TGA and DTA curves in air and argon: (a) for FaAc (1 - air, 2 - argon); (b) for FeAcMTX.

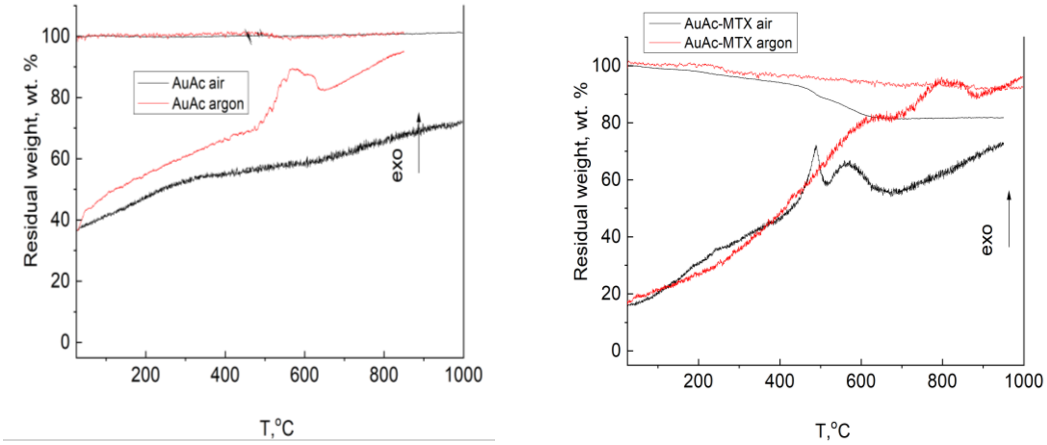

(**a**) (**b**)

Figure S3. TGA and DTA curves in air and argon: (a) for AuAc; (b) for AuAcMTX.

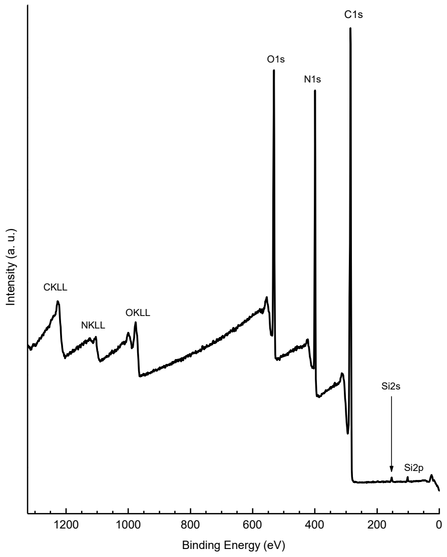

Figure S4. Survey XPS spectra of the MTX sample.

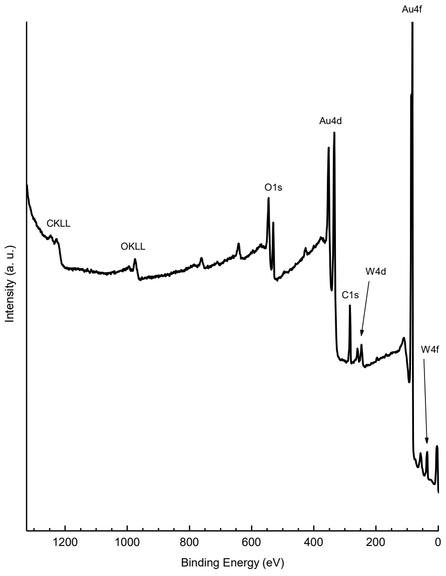

Figure S5. Survey XPS spectra of the AuAc sample.

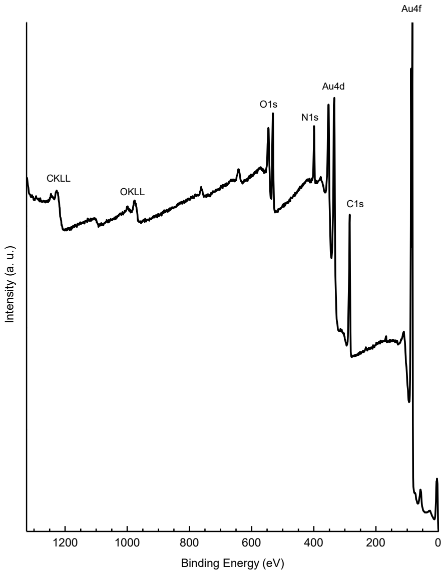

Figure S6. Survey XPS spectra of the AuAcMTX sample.

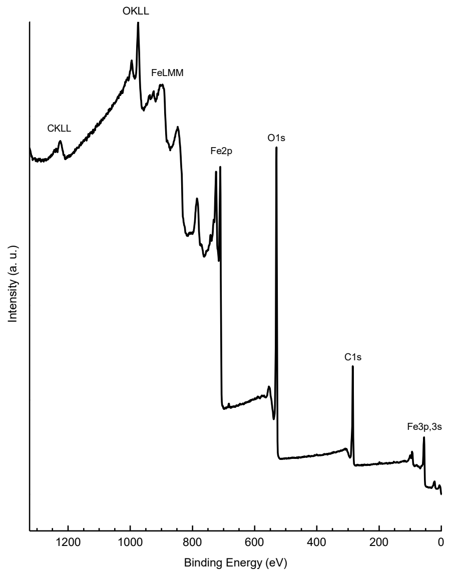

Figure S7. Survey XPS spectra of the FeAc sample.

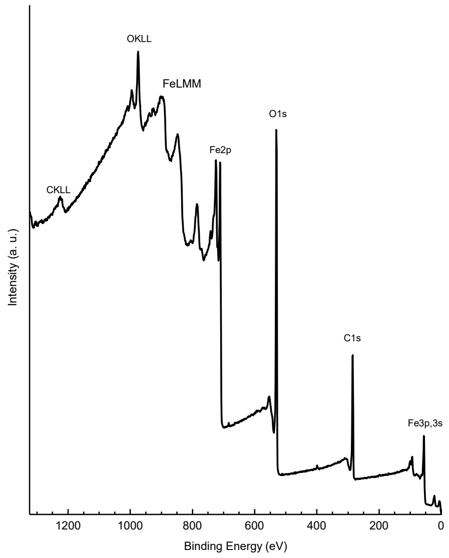

Figure S8. Survey XPS spectra of the FeAcMTX sample.
